# A Novel Role for an ECF Sigma Factor in Fatty Acid Biosynthesis and Membrane Fluidity in *Pseudomonas aeruginosa*

**DOI:** 10.1371/journal.pone.0084775

**Published:** 2013-12-30

**Authors:** Ana Laura Boechat, Gilberto Hideo Kaihami, Mario José Politi, François Lépine, Regina L. Baldini

**Affiliations:** 1 Departamento de Bioquímica, Instituto de Química, Universidade de São Paulo, São Paulo, São Paulo, Brazil; 2 INRS-Institut Armand-Frappier, Université du Québec, Laval, Québec, Canada; Instituto de Biociencias - Universidade de São Paulo, Brazil

## Abstract

Extracytoplasmic function (ECF) sigma factors are members of cell-surface signaling systems, abundant in the opportunistic pathogen *Pseudomonas aeruginosa*. Twenty genes coding for ECF sigma factors are present in *P. aeruginosa* sequenced genomes, most of them being part of TonB systems related to iron uptake. In this work, poorly characterized sigma factors were overexpressed in strain PA14, in an attempt to understand their role in the bacterium´s physiology. Cultures overexpressing SigX displayed a biphasic growth curve, reaching stationary phase earlier than the control strain, followed by subsequent growth resumption. During the first stationary phase, most cells swell and die, but the remaining cells return to the wild type morphology and proceed to a second exponential growth. This is not due to compensatory mutations, since cells recovered from late time points and diluted into fresh medium repeated this behavior. Swollen cells have a more fluid membrane and contain higher amounts of shorter chain fatty acids. A proteomic analysis was performed to identify differentially expressed proteins due to overexpression of *sigX*, revealing the induction of several fatty acid synthesis (FAS) enzymes. Using qRT-PCR, we showed that at least one isoform from each of the FAS pathway enzymes were upregulated at the mRNA level in the SigX overexpressing strain thus pointing to a role for this ECF sigma factor in the FAS regulation in *P. aeruginosa.*

## Introduction

Alternative sigma factors of the extracytoplasmic function (ECF) family are members of cell-surface signaling systems, which also include regulators that modulate ECF sigma function and proteins responsible for sensing environmental cues [1-3]. The cell envelope is in direct contact with the milieu, being the first structure to sense environmental changes, triggering adaptive responses. The effect of variations in the fluidity of cell membranes on the activation of stress responses has been reported for several bacteria (reviewed by Los and Murata [4]), as well as the cells´ response altering the membrane lipid components due to stress conditions (reviewed by Mansilla et al.[5])

*Pseudomonas aeruginosa* is an ubiquitous gammaproteobacterium that can infect phylogenetically distant hosts [6,7]. The presence of a large number of signaling systems in *P. aeruginosa*, including two-component and cell-surface signaling systems, may be responsible for the ability of this bacterium to colonize a broad range of environments and hosts, enabling it to recognize and respond properly to a multitude of challenges [8]. From the twenty genes in the PA14 genome coding for ECF sigma factors, twelve are predicted or proven to be part of systems involved in siderophore uptake, reflecting the importance of iron in the bacterial metabolism [1,9-13]. Among the best studied *P. aeruginosa* ECF sigma factors are AlgU, which regulates alginate production [14]; and PvdS and FpvI, involved in the uptake of the siderophore pyoverdin [15,16], both traits related to virulence and colonization, with extensive literature covering their functional and molecular characterization [10,17-19]. Some other ECF sigma factors, like VreI and SigX have also been subject of investigation and the VreI regulon has been recently described [20-23]. In strain PAO1, SigX was shown to be required for growth under low osmolarity and to activate *oprF* transcription [22]. SigX is an orphan ECF factor, meaning that no anti-sigma factor is encoded in the same operon and none has been described to regulate its activity to date. The closest homologue of SigX that has been functionally analyzed is SigW from *Bacillus subtilis*, with 49% of similarity at the aminoacid level. SigW was shown to regulate membrane fluidity by changing the branched-to-linear fatty acid ratio by altering the trancriptional levels of two genes involved in the fatty acid synthesis (FAS) [24].

**Figure 1:**
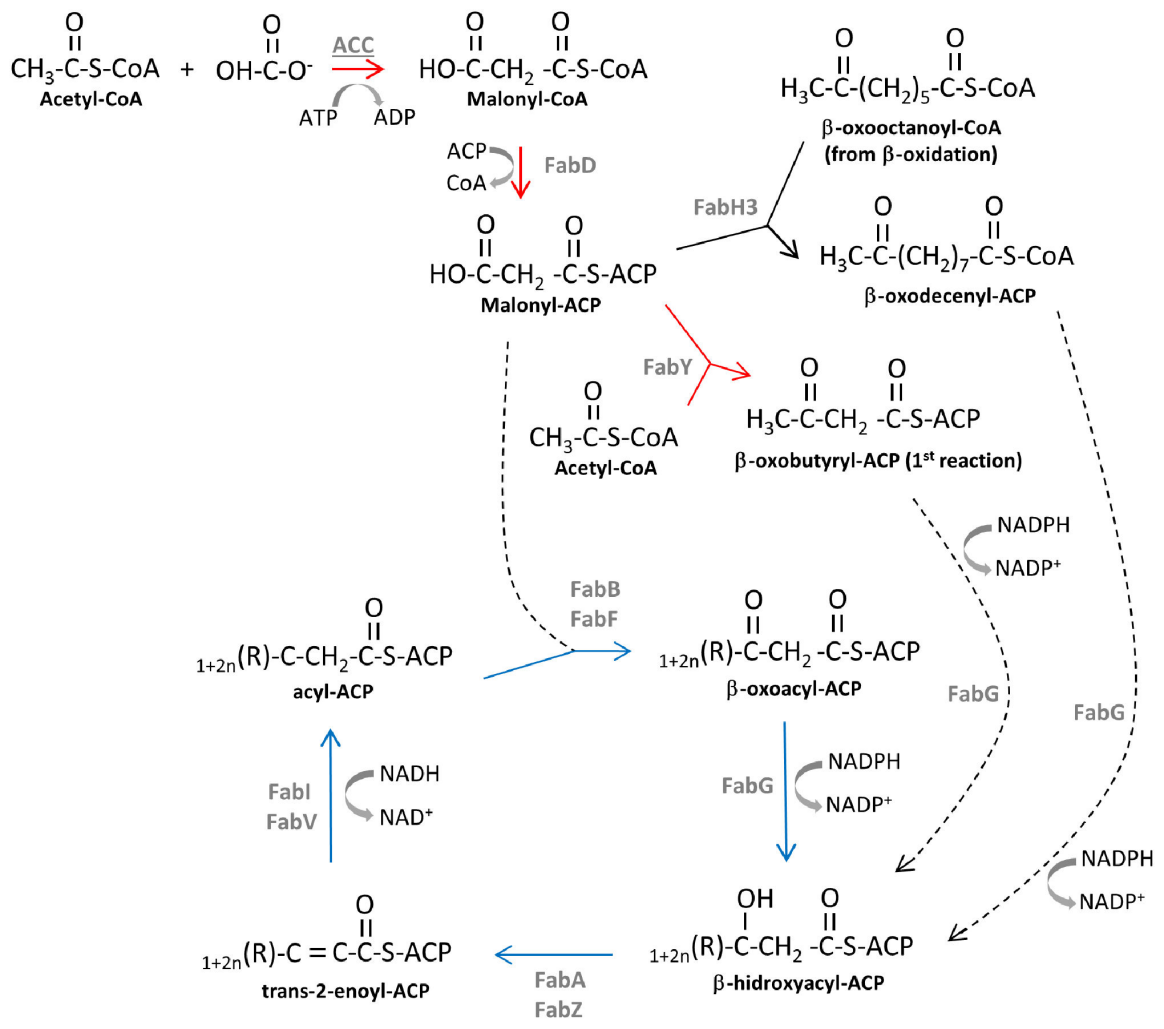
Fatty acid biosynthesis in *P. aeruginosa*. After the initiation of the acyl chain biosynthesis (reactions represented by red arrows), the biosynthetic cycle is repeated until the assembly of C_16_ or C_18_ acyl chains (blue arrows). The dotted arrows represent the entry of compounds in the elongation module.

The initial step in the FAS pathway (Figure 1) is carried out by acetyl-CoA carboxylase (ACC), which catalyzes the ATP-dependent production of malonyl–CoA from acetyl–CoA by adding CO_2_ from bicarbonate. Malonyl–CoA is transferred to the acyl carrier protein (ACP) by FabD (malonyl-CoA-ACP transacylase) [25-27]. In the first condensation reaction, a β-oxoacyl-(ACP)-synthase adds an acetyl-CoA molecule to malonyl-ACP, generating the intermediate β-oxobutyryl-ACP, which is elongated in a cyclic manner to extend the saturated fatty acyl chain by two carbon units in each cycle. In *E. coli*, this step is catalyzed by FabH, but, in *P. aeruginosa*, it was recently shown that FabY acts preferentially in the condensation of acetyl-CoA with malonyl-ACP to initiate the FAS pathway, instead of the well characterized FabH [28]. β-oxoacyl-ACP is reduced in the first reaction of the FAS cycle by FabG (β-oxoacyl-ACP-reductase) in a NADPH-dependent reaction, generating β-hydroxyacyl-ACP, which has its hydroxyl group removed by FabA or FabZ (3-hydroxyacyl-ACP-dehydratases), resulting in a double bond, which is reduced in a NADH-dependent reaction catalyzed by FabI or FabV in *P. aeruginosa* (NADH-dependent enoyl-ACP-reductases). At this point, the cycle begins again with the condensation of acyl-ACP with another malonyl-ACP group, but the successive condensation reactions are catalyzed by either FabB (β-oxoacyl-ACP-synthase I) or FabF (β-oxoacyl-ACP-synthase II) (reviewed by Chan and Vogel [26]) (Figure 1). For the synthesis of unsaturated fatty acids (UFAs), FabA acts also as an isomerase on the trans-2-decenoyl-ACP, generating *cis*-3-decenoyl-ACP that bypasses the FabI/FabV reduction step and can be elongated by FabB [26,29]. Desaturating enzymes DesA and DesBC, absent from *E. coli*, can also generate UFAs in *P. aeruginosa* by introducing a double bond in existing phospholipids or saturated acyl-CoA, respectively, in oxygen-requiring reactions [29,30].

FAS in bacteria is regarded as a housekeeping function and the regulation of the genes involved in the essential steps is not well explored, despite of regulatory systems being described for altering the composition of the lipid content of membranes. In *E. coli*, the regulation of the synthesis of saturated versus unsaturated fatty acids targets *fabA* and *fabB* via FabR, which regulates UFA synthesis by controling *fabA and fabB* transcription positively or negatively, depending on the availability of UFAs. The *E. coli* β-oxydation repressor FadR also acts as a *fabA* and *fabB* activator [31,32]. Recently, a search for *P. aeruginosa fabAB* transcriptional activators was not able to detect functional homologues of FabR and FadR [33], but this operon is repressed by DesT bound to long chain unsaturated acyl-CoA [34], which also represses the *desCB* desaturase genes. In *B. subtilis*, the ECF sigma factor SigW is responsible for activating the expression of *fabF* from a promoter that lies inside the *fabHa* coding sequence upstream, lowering the transcription of *fabHa* and thus favoring the activity of FabHb, which will decrease the branched lipid content. Higher FabF levels also account for longer FA chains and both mechanisms contribute to a more rigid membrane in this Gram-positive bacterium [24]. No global direct positive regulators of FAS genes expression are yet recognized for the model organisms *E. coli*, *P. aeruginosa* nor *B. subtilis.*

In this work, we found that overexpression of the ECF sigma factor SigX results in growth and morphology changes in *P. aeruginosa* PA14, and we show that high levels of SigX favor the upregulation of a set of FAS enzymes, altering lipid composition and membrane fluidity.

## Materials and Methods

### Ethics statement

Animals for antiserum production were handled in strict accordance with good animal practice as defined by the relevant national and local animal welfare bodies. All animal work was approved by the appropriate committee (Comissão de Ética em Experimentação Animal, CEUA, Instituto de Química, Universidade de São Paulo).

### Strains, plasmids, oligonucleotides and culture conditions

*P. aeruginosa* PA14 [35] was used as background for ECF sigma factors overexpression and reporter constructions. The SigX and PA14_21550 overexpression ALB04 and ALB02 strains were obtained by cloning *sigX* or PA14_21550, respectively, under the control of an arabinose inducible promoter in pJN105, a pBBR derivative plasmid carrying a gentamycin resistance cassette [36]. As control, PA14 carrying the pJN105 vector (ALB01) was used. For reporter *lacZ* fusions, the upstream region of the genes in study were cloned in mini-CTX-*lacZ* [37] and β-galactosidase assays were carried out with standard methods [38]. *E. coli* DH5α and S17-1 were used for the constructions and for conjugation with *P. aeruginosa*, respectively. The oligonucleotide sequences of primers used throughout this study are listed in Table S1. Cultures were grown in Luria-Bertani (LB) broth with antibiotics (50 μg/mL gentamycin for *P. aeruginosa* and 12.5 μg/mL or 100 μg/mL tetracyclin for *E. coli* and *P. aeruginosa*, respectively) and 0,2% arabinose, when needed. 

### Growth and survival evaluation

 For assessing both growth and survival, ALB01 and ALB04 were grown from an initial OD_600nm_ of 0.1 in 25 mL LB supplied with gentamycin and arabinose under agitation of 240 rpm at 37°C. OD_600nm_ was measured periodically and serial dilutions were plated in LB-agar. After 16 hours of incubation at 37°C, colonies were counted and the result was plotted as an average of biological triplicates.

 The fluorescence microscopy pictures were obtained after washing ALB01 and ALB04 cells twice with 10mM MgSO_4_ and incubating them with LIVE/DEAD *Bac*Light bacterial viability kit (Invitrogen) according to the manufacturer protocol. A Nikon TE 300 inverted microscope containing the appropriate filters was used and the images were treated with Image J (http://rsb.info.nih.gov/ij/)*.*

For flow cytometry assay, aliquots of 1mL of ALB01 and ALB04 cultures induced with arabinose after three hours of growth were collected at the indicated times and washed twice with 10 mM MgSO_4_. The sample OD_600_ was adjusted to 3.0 and diluted 1:100 (resulting in 1 x 10^5^ cells/mL) before being incubated with 0.5 μL/mL of LIVE/DEAD *Bac*Light bacterial viability kit (Invitrogen) dyes mix. The cells fluorescence were analyzed in a Beckman Coulter FC500 MPL flow cytometer. 

### Anti-SigX polyclonal serum production and SigX immunoblots

*sigX* coding region was cloned under control of an IPTG-induced promoter in pET43-a (EMD Biosciences) to overexpress SigX with N-terminal Nus and His tags in *E. coli* DH5α. An *E. coli* culture growing in 100 mL LB was incubated at 30°C for 6 hours after addition of IPTG to a final concentration of 0.6 mM following harvesting and ressuspension in 10 mL lysis buffer (20 mM sodium phosphate pH 7.4; 500 mM NaCl; 20 mM imidazole; 150 μg/mL of phenylmethylsulfonyl fluoride). The cell suspensions were submitted to four 30 seconds cycles of sonication in an ice bath. Nus-His-SigX was purified using a Ni-NTA column (Invitrogen) and the fraction eluted with 300 mM imidazole was treated with enterokinase (Sigma) (0.02 U/mg), 2mM CaCl_2_ and 1% Tween 20 during 16 hours at 37 °C. The bands corresponding to SigX-His (30.7 KDa) and Nus-tag were resolved in SDS-PAGE and the SigX-His band was cut from the preparative gel, macerated in a potter, mixed with an equal volume of Freund’s adjuvant (Sigma) and inoculated in a rabbit to obtain the anti-SigX polyclonal serum used in immunoblots. 

For immunoblot assays, bacteria were grown in LB containing gentamycin and arabinose, when indicated. Periodically, 1 mL of each culture was centrifuged (13,000 *g*, 2 minutes), suspended in SDS-PAGE loading buffer in a volume normalized by OD_600nm_, boiled during 5 minutes and centrifuged again for 5 minutes. Proteins were separated by electrophoresis on a 15% SDS-PAGE gel and transferred to a nitrocellulose membrane using standard methods. Equal protein amounts were confirmed by Coomassie staining of identically loaded gels run in parallel. Membranes were incubated with α-SigX polyclonal serum in 1:1000 (v/v) in TBS-milk 5%. Secondary detection was carried out with Anti-Rabbit IgG Alkaline Phosphatase Conjugate (Sigma) or CF680 Goat Anti-Rabbit IgG (H+L) (min X Human, Mouse, and Rat) (Uniscience) in TBS-T at the concentrations indicated by the manufacturer protocol and development was performed using standard methods.

### Proteomic analysis

Two proteomic analyses were carried out, as follows. The first experiment was performed with ALB01 and ALB04 grown in LB containing gentamycin for three hours (OD_600nm_ of 1.3 - 1.5), when arabinose was added. These late inductions were done to avoid the drastic differences between ALB01 and ALB04 growth rates. The second experiment was performed with ALB04 alone with addition of arabinose after 1.5 hours of growth (OD_600nm_ of 0.34 - 0.4). In both experiments, after three hours of induction (and after 28 hours for ALB04 only in the second assay), cells were harvested by centrifugation (13,000 x g for 2 minutes) and washed subsequently in 100 mM Tris-HCl and 10 mM Tris-HCl, pH 8.0. Cells were suspended in lysis solution (8M urea, 2M thiourea, 2% CHAPS, 40 mM DTT, and 1 tablet of the Complete Mini Protease Inhibitor Cocktail (Roche) for each 10 mL of solution and sonicated in four cycles of 15 seconds. Protein concentration was determined by the Bradford method [39] and 350 μg of protein in rehydration solution (lysis buffer with 2% Pharmalyte (GE), 10% glycerol, bromophenol blue) in a final volume of 350 μL were incubated with 18 cm pH 3-10 nonlinear strips (GE) during 16 hours at room temperature and submitted to isoeletric focusing in IPGphor III (GE Healthcare). The proteins were further separated on 12% polyacrylamide gels at 10 W for 17 hours. The gels were incubated in a 40% ethanol/10% acetic acid solution, stained with a colloidal Coomassie brilliant blue solution (0.08% Coomassie brilliant blue, 1.6% H_3_HPO_4_, 600 mM (NH_4_)_2_SO_4_; 20% methanol) overnight and destained with water before scanning in the ImageScanner III (GE Healthcare). Statistical treatment of spots using *t* test (p<0.05) was carried out with Delta 2D software (Decodon), considering ratio of mean normalized volumes between samples < 0.7 or > 1.3 as a cutoff. Mass characterization of the trypsin digested proteins from cut spots was done by LC-MS/MS in the Mass Spectrometry Laboratory at the Brazilian Biosciences National Laboratory, CNPEM-ABTLuS. The peptide sequences obtained were used as a query in a BLAST analysis of the P. *aeruginosa* PA14 genome [40].

### β-galactosidase activity assays and quantitative RT-PCR

To confirm the proteomic analysis results of SigX overexpression, ALB01, ALB02 and ALB04 containing the reporter constructs integrated in the chromosome were grown in LB supplemented with gentamycin and arabinose, under the same conditions used for growth determination. To verify a natural induction of *sigX* and of one of its putative targets, strains PA14::*sigX*_*lacZ* and PA14::*fabH3_lacZ* were grown in regular LB with (5 g/L NaCl, which corresponds to 171 mM) or in LB without NaCl. Samples were collected at the time intervals indicated, and the assays performed as described [38]. 

For qRT-PCRs, total RNA was extracted with Trizol (Invitrogen), treated with DNase I (Thermo Scientific) and used for cDNA synthesis with Improm II (Promega) or Superscript III (Invitrogen) and hexamer random primers (Thermo Scientific). The resulting cDNA was amplified with specific primers using Maxima SYBRGreen/ROX qPCR Master Mix (Thermo Scientific) and the 7300 Real Time PCR System (Applied Biosystems). *nadB* was used as internal control for normalization of total RNA levels [41]. The relative efficiency of each primer pair was tested and compared with that of *nadB* and the threshold cycle data analysis (2^-ΔΔCt^) was used [42]. All reactions were performed in triplicates, the assays were repeated at least twice using independent cultures and the results of one representative experiment are shown, with average values of technical triplicates and error bars representing standard deviation of ΔΔCt. The statistical significance of expression ratios was calculated using *T*-test [43], and the results with *p*<0.05 were considered different from the control.

### Search for putative Sig*X* binding sites

The *Consensus* software [44], available at HTTP://rsat.ulb.ac.be/rsat/ was used to search for conserved sequences that could represent SigX binding sites. 500pb regions upstream the translational start site of each of the FAS operons detected as upregulated in the overexpression of SigX were analyzed. 

### Fatty acid composition analysis

The fatty acid composition of whole cells was assessed through fatty acid methyl ester (FAME) analysis. FAMEs were obtained from the whole lyophilized ALB01 and ALB04 cells grown for four hours with arabinose using methanol and sulfuric acid at 100°C for one hour. FAMEs were then extracted and injected (1 µL) in a 30m DB-5 column connected to a mass spectrometer. Naphthalene (retention time RT= 7.84 min) was used as an internal standard in order to quantify the various congeners.

### Steady-state fluorescent anisotropy assay

 The anisotropy of 1,6-diphenyl-1,3,5-hexatriene (DPH) added to the whole cells was determined as reported [45]. Briefly, ALB01 and ALB04 were grown in LB with 0.2% arabinose for four hours. Cell pellets were washed twice with 10mM MgSO_4_ and suspended in the same solution to OD_600nm_ = 0.4. DPH was added to the bacterial solution to a final concentration of 4 x 10^-6^ M and the tubes were incubated for 30 minutes at 37°C before measuring the membrane anisotropy in a DM3000F SPEX fluorimeter equipped with Glan-Thompson polarizers.

 The degree of polarization was determined from measurements of fluorescence intensities parallel and perpendicular with respect to the plane of linearly polarized excitation light. The excitation wavelength was set at 365nm and fluorescence intensities were measured at 425nm parallel (I´_*vv*_) and perpendicular (I´_*vh*_) to the excitation light. Anisotropy (r) was calculated using the intensity before (I^0^_*vv*_ and I^0^VH) and after (I'_*vv*_ and I'_*vh*_) the addition of DPH in accordance with the following equation [46]: 

$$r=\frac{\left(I'vv-I^{0}vv)-G(I'vh-I^{0}vh\right)}{\left(I'vv-I^{0}vv)-2G(I'vh-I^{0}vh\right)}$$

In the above equation, the the *I* ^0^ values correspond to the intrinsic fluorescence of cells and the G factor is the ratio of sensitivities of the detection system for vertically and horizontally polarized light. This correction factor is the ratio of vertical to horizontal fluorescence intensity when the excitation light is polarized at the horizontal position G=I_hv_/I_hh_ [47]. Anisotropies values are related to the lipid bilayer organization or to the local microenvironment fluidity, thus a higher anisotropy value corresponds to a more organized or less fluid lipid bilayer. 

### Temperature stress assays

 For cold shock, ALB01 and ALB04 strains were cultivated for 3 hours under the same conditions described for the growth curve (t=0), a sample was serially diluted and plated for CFU counting and each culture was split in two flasks, one of them remaining at 37°C and the other switched to 15°C. After 2 hours in distinct temperatures, cells were serially diluted, plated and incubated overnight at 37°C for CFU counting. A similar protocol was followed for the heat shock treatment, except that cultures at 30°C were divided and incubated for one hour at 30°C or 42°C. Results are shown as variation in the CFU counts from the t=0 time point for each culture.

## Results

### Sig*X* overexpression has effects on growth and morphology of *P. aeruginosa* PA14

**Figure 2:**
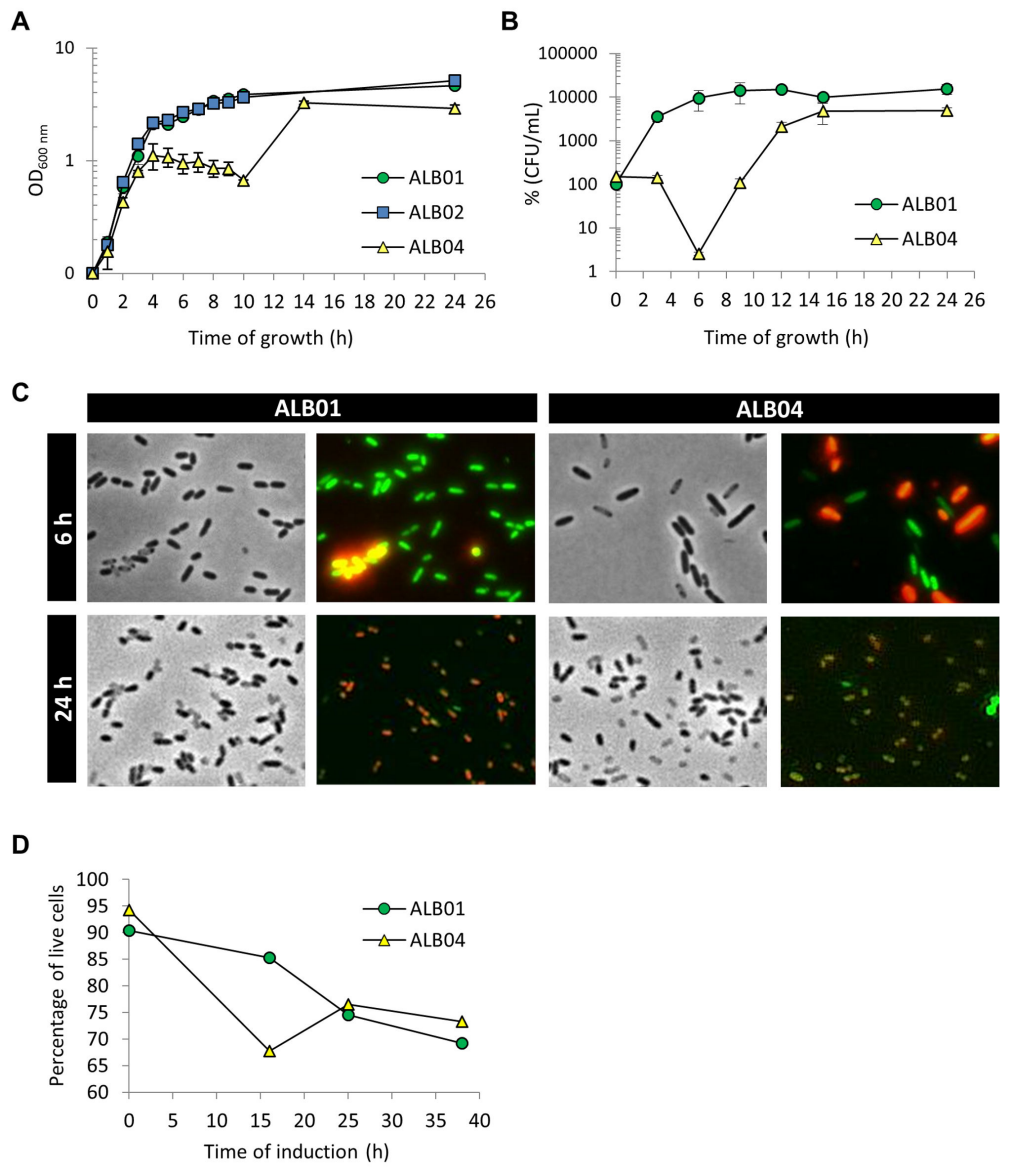
SigX overexpression alters growth and morphology of *P. aeruginosa*. **A**. Growth curve of sig*X* overexpressing strain (ALB04) and control (ALB01) in LB with 50 mg/mL gentamycin and 0.2% of arabinose at 37°C and 240 rpm. **B**. Survival was measured by counting colony forming units (CFU) along the growth curve. **C**. Phase contrast and fluorescence microscopy. In both panels, the column on the left shows the cells as seen in phase contrast microscopy, while the column on the right depicts fluorescence microscopy of the cultures previously stained with LIVE/DEAD BacLight Bacterial Viability Kit (Invitrogen), where membrane damaged cells (dead) are colored in red. All pictures are at the same scale. D. Flow cytometry analysis of LIVE/DEAD stained cells. The values plotted in A and B are means of three biological replicates and error bars represent standard deviation. Flow cytometry was carried out with biological duplicates in two different occasions and values shown are representative of one experiment.

*P. aeruginosa* PA14 overexpressing SigX (ALB04) from an arabinose inducible promoter showed a biphasic growth curve, in which the stationary phase occurs earlier than in the control strain (PA14 carrying the vector alone, ALB01) (Figure 2A). This first phase of apparent growth arrest, that lasts around six hours at 37°C, is followed by a second burst of growth, reaching a second stationary phase. In fact, when we analyze the CFU counts during this first growth arrest phase, we observe that cells lose viability, indicating cell death, but the remaining cells can recover growth at later time points (Figure 2B, D). The growth resumption is not due to a suppressor mutation, since a sample of the culture in late stationary phase diluted in fresh medium behaves in the same manner, repeating the biphasic curve (not shown). When cells collected during the first stationary phase (6h) were observed by phase contrast microscopy, ALB04 cells were swollen in comparison with the control cells at the same time point (Figure 2C). Concomitantly, a LIVE/DEAD assay was performed, showing that most of those swollen cells are dead, but some remain viable, as confirmed using flow cytometry analysis (Figure 2C, D). When ALB04 cultures are in the second stationary phase, after growth recovery (24h), cells are alive and display regular size, whereas the control culture incubated simultaneously consists of mostly dead cells, typical of late stationary phase (Figure 2C, D). 

These data are indeed a specific result of SigX overexpression and not a general stress following overexpression of any given ECF sigma factor, since overexpression of other five ECFs, annotated as PA14_21550 (Figure 2A, strain ALB02), PA14_26600, PA14_28970, PA14_46810 and VreI (not shown), did not result in any alteration in the growth curve and cellular morphology. 

### Sig*X* protein levels drop down following its overexpression

**Figure 3:**
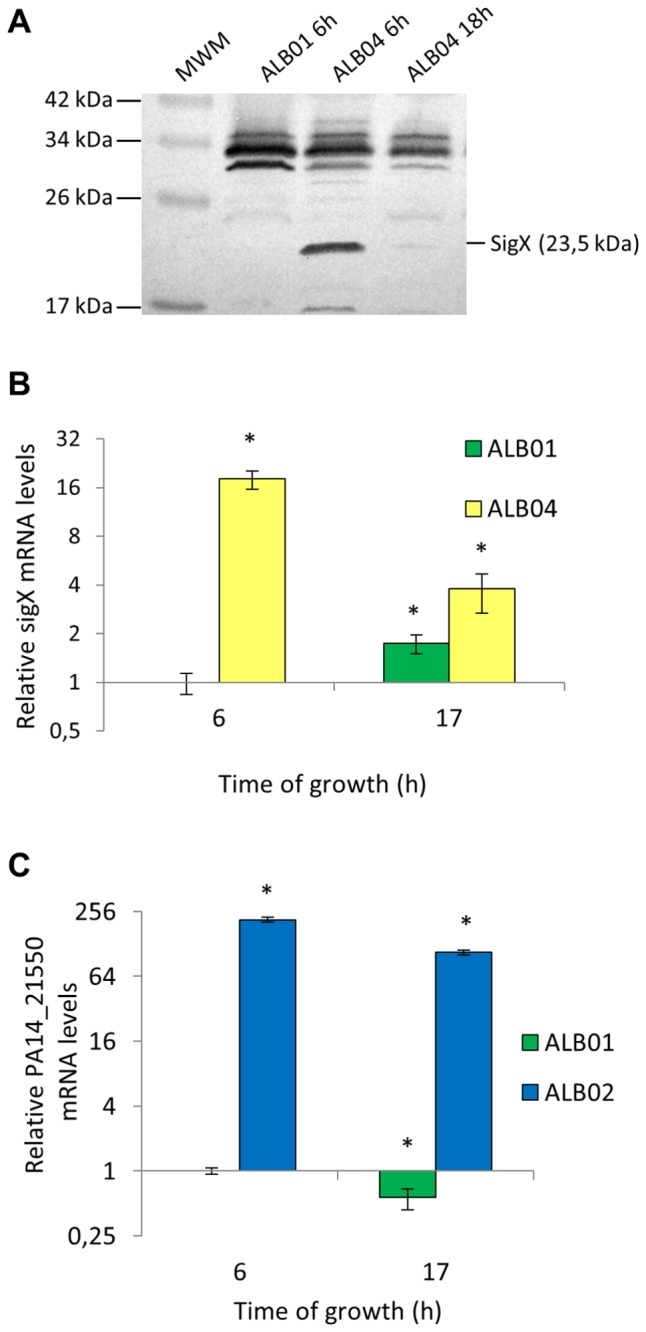
SigX levels are controlled at protein and mRNA levels in the overexpression strain ALB04. **A**. Immunobloting using a polyclonal anti-SigX antibody and total protein extracts obtained from cultures growing in the presence of arabinose and gentamycin. The Spectra Multicolor Broad Range Protein Ladder (Fermentas) was used as molecular weight marker (MWM). **B** and **C**. Quantitative RT-PCR using cDNA made by reverse transcription of total RNA from ALB01 (control), ALB04 (sig*X* overexpression) or ALB02 (PA14_21550 overexpression) cells growing in inducing conditions and specific sigX (B) or PA14_21550 (C) primers. Asterisks indicate significant differences comparing to the reference sample ALB01 6h, plotted as 1 (*p*<0.01).

To evaluate the amount of the SigX protein in ALB01 and in the *sigX* overexpressing strain ALB04 along the growth curve, SigX immunobloting was performed with proteins collected after 6 and 18 hours of growth in the presence of arabinose. The method was not able to detect wild-type levels of SigX in the control strain ALB01, but a band corresponding to SigX is present after 6 hours of overexpression in ALB04 and fades during the second exponential phase (Figure 3A). The overexpression plasmid is maintained through antibiotic pressure and arabinose is not consumed, therefore it was expected that the SigX protein amount would be constant along the growth curve. The levels of *sigX* mRNA in ALB04 agree with the protein expression profile, decreasing 4.7-fold at later time points (Figure 3B). In the wild type background (ALB01), *sigX* mRNA levels increased slightly (1.7-fold after 17h of growth, *p*<0.01) at stationary phase (Figure 3B). Therefore, while *sigX* levels increase at late time points in the control strain ALB01, it decreases in the overexpressing strain ALB04.

In order to verify whether the same trend would be observed with other ECF sigma factor, what would suggest a general effect of decrease in the expression levels of a regulatory gene after its artificial induction, strain ALB02 was used. This strain overexpresses the PA14_21550 gene and was constructed with same strategy as ALB04. In the overexpressing PA14_21550 strain ALB02, there was a remarkable increase in PA14_21550 mRNA levels at 6h of growth in the presence of arabinose, as compared to the control strain ALB01 (Figure 3C). Despite this high level of overexpression at 6h, after 17h of induction, PA14_21550 mRNA levels drop to half of the previous amount (200- to 90-fold, as compared to the control strain ALB01), which is roughly the same rate of decrease of PA14_21550 mRNA levels in the control strain ALB01 (from 1 to 0.56) (Figure 3C). This set of data may suggest that the biphasic growth curve in the *sigX* overexpressing strain ALB04 might be correlated to the drop in the SigX amount along the time course. 

### Sig*X* regulates expression of fatty acid biosynthesis enzymes

 In an attempt to understand the growth arrest and swelling of the cells overexpressing SigX, a proteomic analysis was performed with total protein extracts of ALB04 induced cells (3 hours) compared to ALB01 in the same conditions. Recovering ALB04 cultures (28 hours of induction) were also compared to ALB04 after 3 hours of induction (Tables 1 and S2). Statistical analysis detected more than a hundred spots differentially expressed and many of those could be easily noted from visual observation of the gels (Figure S1). From 85 spots recovered, a total of 44 were successfully identified and special attention was given to 26 spots that were more intense in SigX overexpressing cells (ALB04 at 3 hours of arabinose induction) and which showed a reduction in recovered cells (ALB04 at 28 hours of arabinose induction), following SigX decay. In this context, at least seven Fab enzymes of the FAS pathway (FabD, FabY, FabH3, FabG, FabG2, FabV and FabF2, Table 1) were induced comparing ALB04 to ALB01 at the first time point, in addition to three subunits of acetyl-coA carboxylase (ACC), the first enzyme of this pathway (Figure 1). FabB, which was induced in SigX overexpressing cells at 3h but was not repressed in recovering cells, was also considered in our analysis, because of its role in FAS. Together with FabA, FabB is the enzyme responsible for anaerobic UFAs synthesis in Gram-negative bacteria [48,49]. This set of data is a strong evidence of SigX involvement in fatty acid biosynthesis regulation. 

**Table 1:** Fatty acid biosynthesis and other selected proteins differentially expressed in ALB04.

**Protein**	**Function^*a*^**	**ALB04 3h/ ALB01 3h^*b*^**	**ALB04 28h/ ALB01 3h^*c*^**
**Fatty acid biosynthesis**			
PA14_01240	carbonic anhydrase	5.77	ND
PA14_17270 (AccA)	acetyl-CoA carboxylase carboxyltransferase subunit alpha	1.98	ND
PA14_64100 (AccB)	acetyl-CoA carboxylase biotin carboxyl carrier protein subunit	4.17	ND
PA14_23860 (AccD)	acetyl-CoA carboxylase subunit beta	2.12	ND
PA14_25650 (FabD)	malonyl-CoA-[acyl-carrier-protein] transacylase	6.42	2.40
PA14_68360 (FabY)	β-oxoacyl synthase I/II	4.19	0.28
PA14_25660 (FabG)	β-oxoacyl-(acyl-carrier-protein) reductase	3.30	0.63
PA14_57050 (FabG2)	β-oxoacyl-(acyl-carrier-protein) reductase	6.80	0.78
PA14_25900 (FabV)	trans-2-enoyl-CoA reductase	5.24	0.76
PA14_21540 (FabH3)	β-oxoacyl-(acyl carrier protein) synthase III	4.12	1.19
PA14_43690 (FabB)	β-oxoacyl -(acyl carrier protein) synthase I	1.69	1.95
PA14_46490 (FabF2)	β-oxoacyl-(acyl carrier protein) synthase II	5.12	1.86
**Other**			
PA14_41575 (SigX)	RNA polymerase sigma factor SigX	22.72	1.88
PA14_17310 (KdsA)	2-dehydro-3-deoxyphosphooctonate aldolase (LPS synthesis)	6.14	2.08
PA14_05460 (BioA)	adenosylmethionine-8-amino-7-oxononanoate transaminase (cofactor synthesis)	4.19	ND
PA14_12130 (Lis)	lipoyl synthase (cofactor synthesis)	2.09	0.42
PA14_63250 (YcfY)	acetyl-CoA acetyltransferase (FA degradation)	1.18	3.46
PA14_62130 (IlvC)	ketol-acid reductoisomerase (CoA synthesis)	1.11	1.44
PA14_41570 (OprF)	major porin and structural outer membrane porin OprF	0.77	4.40

^a^ As annotated [8,40,82]

^b^ fold change in protein amounts in SigX overexpressing cells after 3h of arabinose induction in comparison to the control strain.

^c^ fold change in protein amounts in recovered cells (ALB04 after 28h of arabinose induction)in comparison to control cells (ALB01) after 3h of arabinose induction.

**Figure 4:**
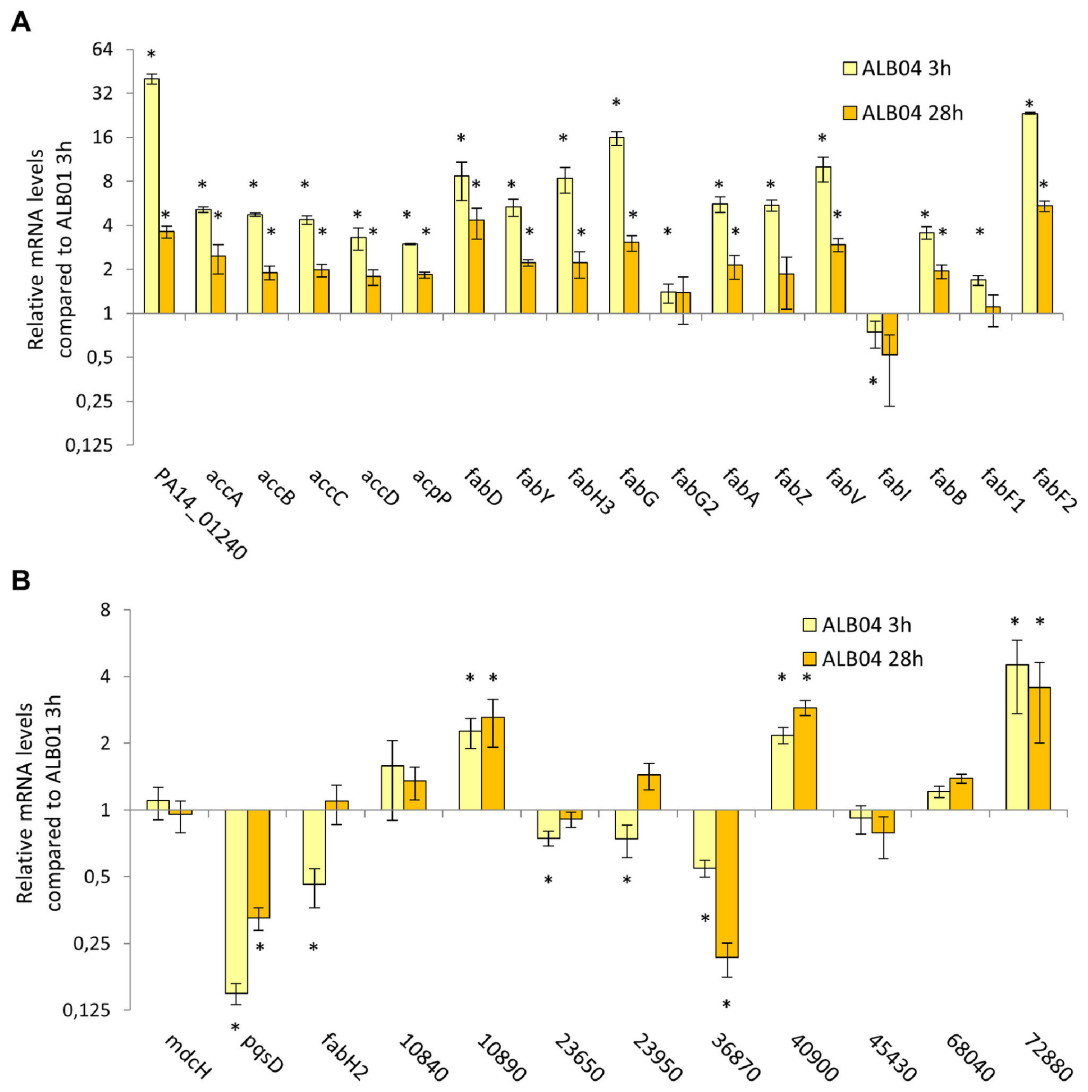
SigX overexpression effect in FAS genes and their paralogues. Comprehensive quantitative RT-PCR assay with cDNA synthesized from RNA of the same cultures used for proteomic analysis shows the effect of SigX overexpression on genes coding for fatty acid biosynthesis enzymes (**A**) and their homologues (**B**). Results are shown as relative values in comparison to the control strain ALB01 at 3h after arabinose induction, considered as 1 and coinciding with the x axis. Significant differences among the values for each gene are shown with an asterisk.

Additional proteins induced in SigX overexpression act directly or indirectly on fatty acids metabolism or in other pathways in which lipids are involved. For example, carbonic anhydrase, which was induced in the proteomic analysis and presented 40-fold higher mRNA levels than in ALB01 (Figure 4A), produces bicarbonate, one of the ACC substrates. Furthermore, adenosylmethionine-8-amino-7-oxononanoate transaminase is involved in the production of biotin, the ACC cofactor. KdsA is coded by an operon responsible for biosynthesis of 3-deoxy-D-manno-octulosonicacid (Kdo) [50], the first sugar of the lipopolysaccharide inner core region, which is crucial for the integrity of the outer membrane [51-53]. LipA catalyzes the radical-mediated insertion of two sulfur atoms into an acyl carrier protein (ACP) bound to an octanoyl group to produce a lipoyl group, participating in lipoic acid metabolism and several other metabolic pathways [54,55]. 

Even though the proteomic analysis showed a clear induction of FAS enzymes, not all enzymes known to be involved in this pathway were detected with this approach. Therefore, we carried out a comprehensive quantitative RT-PCR of all annotated FAS genes in the PA14 genome and some homologues, in order to have a set of differentially expressed FAS genes due to SigX overexpression (Figure 4). 

 All of the genes coding for FAS enzymes detected in the proteomic analysis, namely *accA, accB, accD, fabD, fabY, fabH3, fabG, fabV, fabB* and *fabF2* were also upregulated in the qRT-PCR assays, with higher mRNA levels ranging from 3.6- (*fabB*) to 23.4-fold (*fabF2*) at 3h of induction and relative mRNA amounts decreasing to near the uninduced levels in the recovered cells. The only exception was *fabG2*, with a low level of induction (1.4-fold) at both time points. PA14_01240 (carbonic anhydrase) mRNA levels showed the highest level of induction (40-fold at 3h), and the acyl carrier protein mRNA was also increased (Figure 4A).

 For the FAS genes not detected in the proteomic analysis, we found that *accC, fabA* and *fabZ* followed the same pattern described above. *fabI* and *fabF1* were not regulated by overexpression of SigX. Contrastingly, the uncharacterized *fabH2* gene (PA14_20950/PA3333) seemed to be downregulated at 3h of SigX induction and its mRNA increased to the wild-type levels in the recovering cells (Figure 4A).

*P. aeruginosa* genome has many genes with similarity to those coding for FAS enzymes. *pqsD*, for example, is a *fabH* homologue involved in the synthesis of the *Pseudomonas* quinolone signal PQS. *pqsD* mRNA levels decrease in ALB04 cells, therefore SigX is not involved in the activation of this pathway. *mdcH*, the gene coding for the malonate decarboxylase epsilon subunit, whose product presents 37.4% amino acid identity with FabD, is not induced by SigX overexpression. Several genes for short-chain-dehydrogenases with identities ranging from 24.51 to 31.92% with FabI were also analysed by qRT-PCR and three out of nine presented higher mRNA levels in SigX overexpression and one had lower levels due to SigX overexpression (Figure 4B).

**Figure 5:**
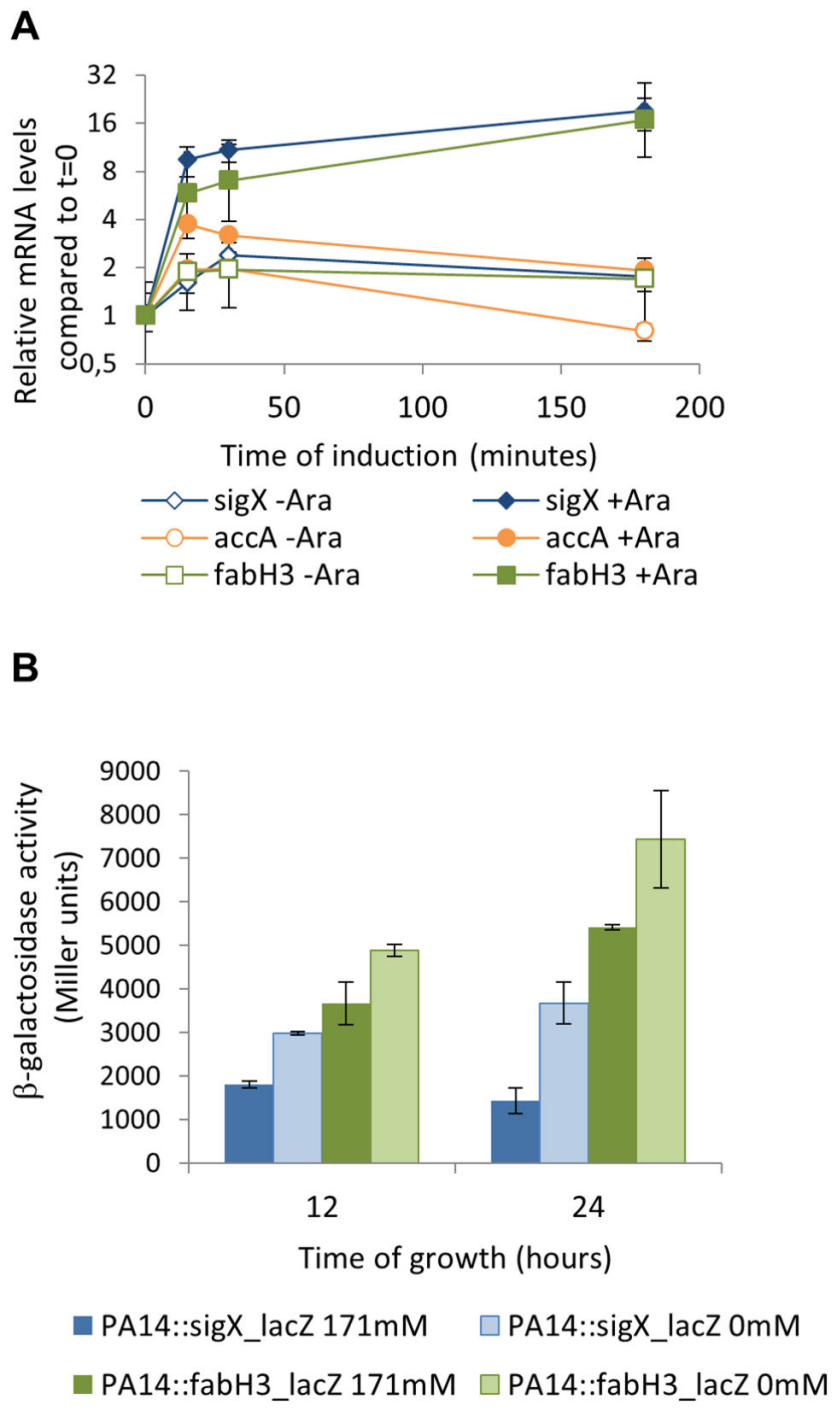
Lower levels of overexpression and natural induction of sig*X* induce target genes. **A**. Arabinose was added to ALB04 cultures at exponential phase and samples (+Ara) were collected after 15, 30 and 180 minutes for RNA extraction and qRT-PCR of sig*X*, accA and *fabH3*. Control samples were taken at the same time points (-Ara). All values are shown as relative to t=0 for each treatment, set as 1. **B**. β-galactosidase activity assay of sig*X* and *fabH3*-*lacZ* promoter fusions in the wild-type background (PA14::sig*X*_*lacZ* and PA14::*fabH3_lacZ*) in LB with 171 mM NaCl or without NaCl. The values plotted are averages of biological triplicates and error bars represent standard deviations.

 All the qRT-PCR and proteomic assays mentioned above were done with cultures growing in the presence of arabinose for 3h, which could lead to other alterations in the cells. To confirm that the increase in expression of selected genes was due to SigX even in lower levels of overexpression, mRNA levels of *accA* and *fabH3* were measured after 15 and 30 minutes of arabinose addition to cultures in exponential growth. No differences in the cell morphology or growth arrest were observed after 30 min of arabinose induction. In these conditions, both *accA* and *fabH3* mRNA levels increased following overexpression of SigX (Figure 5A). As low-salt medium was described as an inducer of SigX in strain PAO1 [22,23], *sigX*- and *fabH3*-*lacZ* promoter fusions were constructed and integrated into PA14 chromosome. In LB without NaCl, β-galactosidase activity was increased about two-fold for each promoter (Figure 5B). Thus, genes that presented increased expression in high levels of SigX overexpression are also induced in lower levels of SigX overexpression and in a condition in which SigX is naturally activated.

 No discernible consensus sequences could be found in the upstream sequences of the FAS operons induced in SigX overexpression that might indicate the binding of an ECF sigma factor. This could be an indication that SigX regulates those operons indirectly and intermediate steps are needed between SigX activation and induction of the target genes described here.

### Proteins not related to FAS are also differentially expressed due to Sig*X* overexpression

 Together with the enzymes related to FAS and related pathways, at least 20 other proteins were also identified in the proteomic analysis (Table S2). Among them, two enzymes implicated for pyocyanin synthesis, PhzD and PhzF, were downregulated after 3h of SigX overexpression and upregulated in the recovering phase. This fact agrees with the observation of the ALB04 cultures in LB with arabinose, which do not present the typical greenish-blue color of *P. aeruginosa* until the growth is resumed at later time points. 

 Four proteins coded by a gene cluster (operons *sucAB-lpdG* and *sucCD*) were also upregulated in ALB04 after 3h of induction, and they are part of the tricarboxylic acid cycle, converting 2-oxo-glutarate in succinyl-CoA. Other induced protein in ALB04 in comparison to the control ALB01 was the cysteine synthase A, encoded by *cysK*. Several of the FAS enzymes induced contain cysteine in their primary sequences, but at this point we cannot correlate such high levels of cysteine synthase with FAS. Translation and protein modification proteins were also differentially expressed in ALB04, some of them with higher levels at 3h of arabinose induction and downregulated at the later time point (such as the ClpP protease and the elongation factor EF-P), and other being inversely regulated (e.g. the GroEL chaperone, a ribosomal methyltransferase and the peroxyredoxin Ahp). Those proteins may have a more general function in dealing with the stress resulting from SigX overexpression and allowing the cells to regain growth. 

### Sig*X* controls transcription of an operon containing genes for a FAS enzyme and the PA14_21550 ECF sigma factor

**Figure 6:**
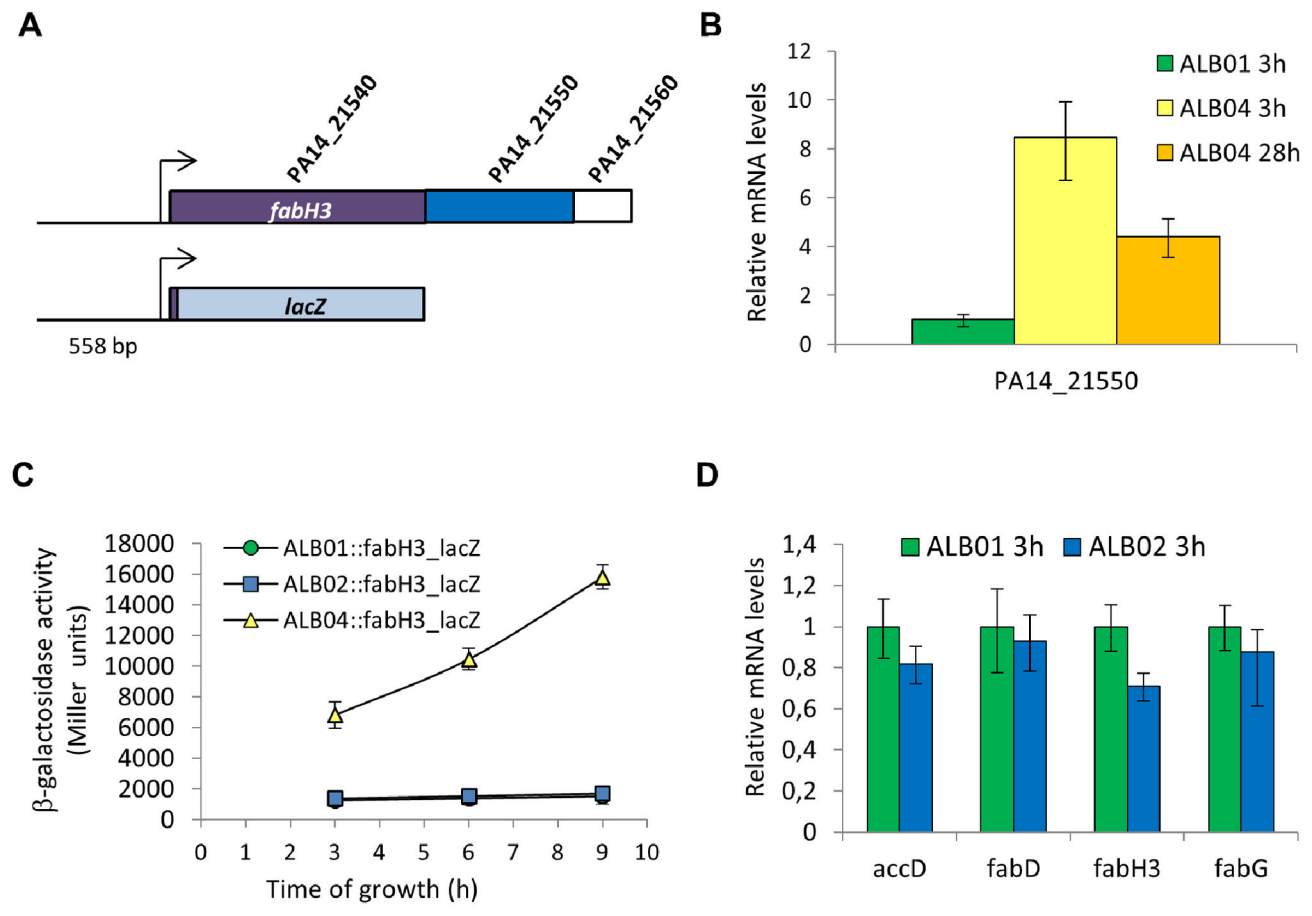
PA14_21550 is induced by sig*X* overexpression, but it does not activate FAS genes. **A**. Genomic region containing *fabH3* and a sketch showing the P*fabH3-lacZ* fusion. **B**. qRT-PCR of PA14_21550 in sig*X* overexpression (ALB04) and control (ALB01) strains. **C**. β-galactosidase activity assay of *fabH3* promoter-*lacZ* fusion in strains overexpressing PA14_21550 (ALB02*::fabH3_lacZ*) and sig*X* (ALB04*::fabH3_lacZ*). The values plotted are averages of biological triplicates and error bars represent standard deviations. **D**. qRT-PCR of some sig*X* overexpression targets in PA14_21550 overexpression strain (ALB02). The values shown in B and D are averages of technical triplicates of at least two independent assays and error bars represent standard deviations.

The *fabH* gene induced by SigX and referred here as *fabH3* (after Zhang et al. [56]) is predicted to be in an operon with genes coding for the putative ECF sigma factor PA14_21550 and a putative anti-sigma, PA14_21560 (Figure 5A). PA14_21550 mRNA levels are also increased in ALB04 overexpression, decreasing in the recovering cells (Figure 6B). Induction of the *fabH3-*PA14_21550*-*PA14_21560 operon by SigX overexpression at the transcriptional level was confirmed using a *lacZ* transcriptional fusion to the region upstream *fabH3* (Figure 6C). This could be indicative of a sigma factor regulatory cascade, with SigX acting indirectly in the lipid biosynthesis genes through PA14_21550. 

### The putative ECF sigma factor PA14_21550 is not responsible for the changes caused by sig*X* overexpression

Because of induction of the PA14_21550 gene in the strain overexpressing SigX, we wondered whether the positive effect observed in FAS genes would be due to this uncharacterized ECF sigma factor and not because of SigX itself. mRNA levels of *fabH3, accD, fabD* and *fabG* are not induced by PA14_21550 overexpression in strain ALB02 (Figure 6D), and the activity of the *fabH3-lacZ* fusion was not altered as well (Figure 5C). PA14_21550 overexpression did not result in cell death (Figure 2A) or swelling (not shown), confirming that both increase in FAS enzymes and alterations in cell morphology and growth are a result of SigX function, and not of PA14_21550. 

### Lipid composition and membrane fluidity of ALB01 and ALB04

**Figure 7:**
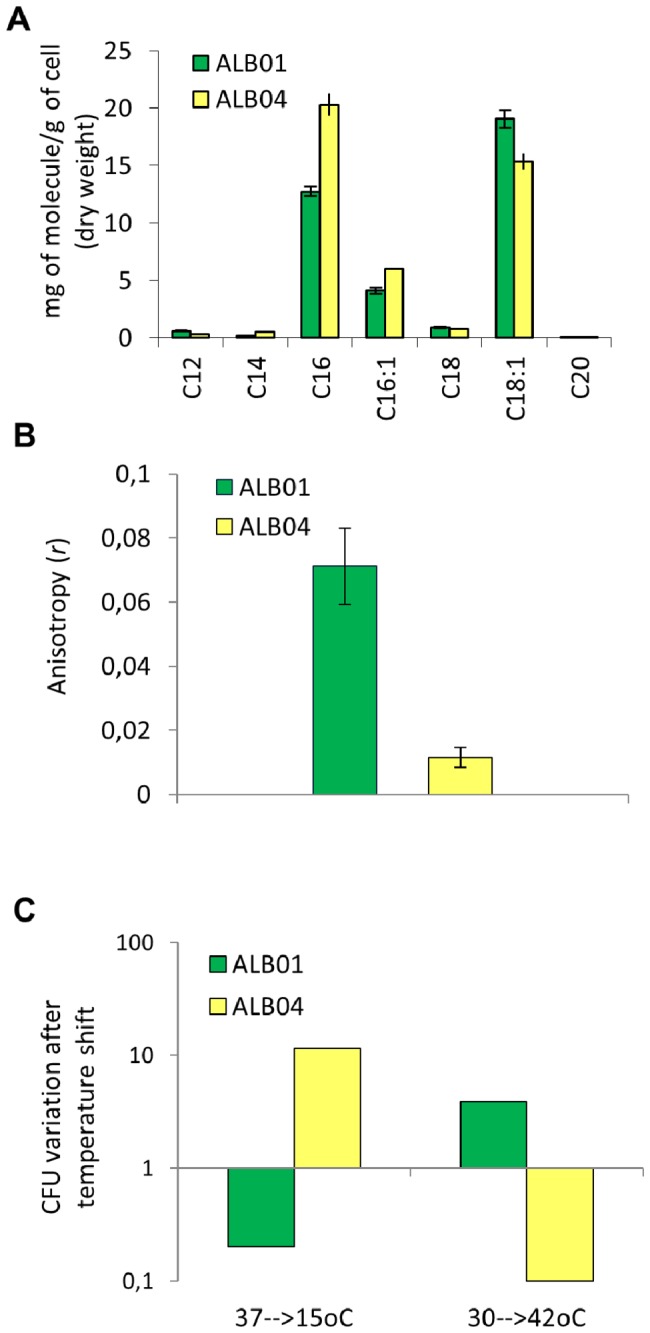
Differences in lipid composition, membrane fluidity and response to temperature stresses in sig*X* overexpressing cells versus control. **A**. Gas chromatography/mass spectrometry analysis of fatty acids methyl esters (FAMEs) derived from ALB01 or ALB04 whole cells preparation. B. Anisotropy assay, where the lower value corresponds to a less organized membrane, so to a higher fluidity. **C**. Cold and heat shock survival. Cultures growing for 3h at 37°C or 30°C were split and a portion of them was shifted to 15°C or 42°C for 2 and 1h, respectively, while another remained at the initial temperature for control. The values are plotted as a ratio of CFU counts of cultures at the stress temperature and the control.

 Analysis of the fatty acids of ALB01 and ALB04 cultures grown in the presence of arabinose during four hours, when a difference in the growth curves is already noted but most ALB04 cells are still alive, showed that hexadecanoic (C16) and octadecenoic acids (C18:1) were the two most abundant compounds in both cultures, with the former being more abundant in ALB04 than in ALB01 (Figure 7A). Smaller amounts of hexadecenoic acid (C16:1) were also detected in both cultures, in a slightly larger proportion in ALB04 than ALB01 (Figure 7A). Thus, ALB04 is enriched with shorter fatty acids when compared to ALB01 (with a 16C:18C ratio of 1.63 for ALB04 and 0.84 for ALB01). ALB04 cells at the same time point presented smaller anisotropy (Figure 7B), indicating that they have more fluid membrane(s) than ALB01, which is in agreement with the higher percentage of shorter fatty acid chains when *sigX* is overexpressed. Together, those findings confirm that *sigX* overexpression has a role in altering membrane fluidity by the activation of *fab* genes, maybe as a result of *fabV* induction, as discussed below. The total lipid content of ALB04 cells was only slightly higher than ALB01´s (about 15%) after 4 hours of arabinose induction, even though most of the FAS genes were upregulated more than 2-fold, indicating that the overall lipid homeostasis was not affected by *sigX* overexpression at this time point.

 Since it is well established that bacteria respond to temperature stresses by adjusting the membrane lipid composition, we tested whether the altered membrane properties of ALB04 in inducing conditions would affect its survival after a temperature shift. Indeed, we found that ALB04 is more resistant to cold shock than ALB01, and the opposite effect was observed when the cultures were submitted to a upshift in temperature from 30 to 42°C (Figure 7C), meaning that ALB04 is more sensitive to heat shock, probably due to its lower membrane organization.

## Discussion

 Fatty acids are essential components of membranes and are important for energy storage. They are also incorporated in coenzymes, quorum sensing signal molecules, siderophores and biosurfactants, making this class of compounds essential for most cellular processes. FAS is a central metabolic pathway to all organisms but two different biosynthetic systems are responsible for the assembly of these molecules. Plants and most bacteria [57,58], generally utilize FAS II, which is composed of multiple discrete enzymes, for *de novo* fatty acid production [59-61]. In contrast, mammals and yeast utilize FAS I, in which the individual functions involved in cytosolic fatty acid synthesis are performed by multiple domains on one or two discrete polypeptide chains [62-64].

FAS II provides flexibility to bacteria, which are more versatile in assembling various fatty acids with different composition and structure, whereas organisms carrying FAS I are limited mostly to long chain saturated fatty acids [61]. As *P. aeruginosa* is a versatile organism adapted to multiple environments and with sophisticated mechanisms of cell-cell communication, it is not surprising that several of the FAS enzymes are encoded by more than one gene and that differences are emerging in respect to the function or specificity of those enzymes in comparison with the most studied *E. coli* FAS pathway [56]. 

 Adjusting the lipid composition of membranes is a common mechanism of cell adaptation to a changing environment. In *P. aeruginosa*, inactivation of *lptA* (coding for a lysophosphatidic acid acyltransferase) affects the fatty acid composition of the membrane phospholipids by increasing the proportion of longer-chain (C18) fatty acids which decreases membrane fluidity. Such a change is sufficient to activate the expression of a number of genes, including *relA* and those involved in quorum sensing, resulting in production of acyl-homoserine lactones (AHL) quorum sensing molecules, independently of cell density, external stress exposure or nutrient starvation [65]. It is long known that *P. aeruginosa* and other Gram-negative bacteria can modulate the saturated/unsaturated FA ratio upon changing the temperature during growth (reviewed in Dubois-Brissonnet et al. [66]). In *E. coli*, the expression of many regulatory genes, like the flagellar master operon and the antisense RNA *micF*, which regulates the expression of the major porin OmpF, is affected by changes in membrane phospholipid composition [67]. *B. subtilis* SigW alters membrane fluidity by regulating genes that favor a less fluid membrane [24]. 

 In this work, we overexpressed putative ECF sigma factors in *P. aeruginosa* PA14 as a first approach to understand their role in the physiology of this bacterium. The striking effect on growth and cell morphology of SigX overexpression led us to focus our study on this particular ECF, which shares 49% similarity with *B. subtilis* SigW, induced by factors that affect cell wall biosynthesis [68] and that belongs to the ECF01 subfamily as proposed by Staron et al. [69]. SigX was previously reported to activate *oprF* expression in PAO1 [22,23]. OprF is the major unspecific porin of *P. aeruginosa*, which plays a role in the maintenance of cell shape and is required for growth in low osmolarity medium [70-72]. Surprisingly, in our proteomic analysis, OprF was not induced in the early growth phase of cells overexpressing SigX (Table 1), excluding the upregulation of OprF as the cause for the altered phenotype observed in ALB04. Moreover, OprF was found to be slightly induced in recovering ALB04 cells (Table 1), when SigX is barely detectable, suggesting a negative role of SigX overexpression on OprF at the protein level in PA14. We have tried by several methods to construct a PA14 *sigX* mutant strain to confirm this seemingly unexpected dissimilarity between PA14 and PAO1 in *oprF* regulation, but all of our attempts have failed, which may indicate variable functions of this ECF sigma factor in distinct strains. No transposon mutant is available for *sigX* in the PA14NR library [73] and overexpression of PA14 *sigX* in PAO1 results in less accentuated changes in the growth curve (not shown). 

 The major alteration in our proteomic profile for cells overexpressing SigX was a high induction of several FAS enzymes, with some differentially expressed spots readily visible to the naked eye (Table 1 and Figure S1). This result prompted us to analyze the mRNA levels for these proteins, as well as for other FAS genes not detected by the proteomic approach, which we acknowledge that is not absolutely quantitative and that several targets of SigX overexpression could be missed due to methodological limitations. Enzymes responsible for all FAS steps were upregulated in ALB04, including acetyl-CoA carboxylase (ACC), FabD, FabH3, FabY, two FabG isoforms (FabG in mRNA and protein levels and FabG2 in protein levels only), FabA, FabZ, FabV, FabB and one isoform of FabF (FabF2), as well as the carbonic anhydrase and the acyl-carrier protein ACP (Table 1 and Figure 4A). Although the overall FAS system in *P. aeruginosa* is very similar to *E. coli*, a higher number of FAS genes is present in the P. *aeruginosa* larger genome and some particularities are emerging as the FAS system of this opportunistic pathogen is being explored in more detail [28,74,75], reflecting its versatile lifestyle. 

*fabD*, coding for a malonyl-CoA-ACP transacilase and induced during SigX overexpression, is the first gene in an operon with one of the *fabG* paralogues [76]. To our knowledge, this *fabG* locus (PA14_25660/PA2967 in PAO1) in the *fabDG* operon, coding for an isoform of oxoacyl-ACP reductase was the only one that had its product characterized *in vitro* [76,77]. Lack of transposon insertions in this locus in the PA14NR and PAO1 libraries [73,78,79] and a report where attempts to create a *fabG* (PA14_25660) disruption failed [76] suggest that this gene product would act as the constitutive reductase. Another FabG isoform, coded by PA14_57050 (PA4389 in PAO1) and here denominated FabG2, was also found as an upregulated protein in the SigX overexpression, but a qRT-PCR analysis did not detect any regulation at the mRNA level. 

In *E. coli*, the initial step of malonyl-ACP and acetylCoA condensation is performed by the acyl-ACP-synthase III FabH [80]. Recently, Yuan et al [75] described FabY, a novel unrelated β-oxoacyl-synthase which catalyzes this reaction in *P. aeruginosa*, and showed that a mutant lacking all four FabH paralogues is viable. Two of those paralogues are coded by *pqsC* and *pqsD* genes in the operon responsible for the synthesis of the Pseudomonas quinolone signal (PQS). PqsC lacks the amino acid residues essential for catalysis and PqsD acts only on PQS synthesis. FabH2 role is currently unknown, but it is encoded by a gene that is part of a long operon with genes coding for an ACP, a non-ribosomal peptide synthase and other products, such as a putative short-chain dehydrogenase and a monooxygenase, that may suggest its involvement in the synthesis of a secondary metabolite. FabH3 uses only β-oxooctanoyl-CoA obtained from β-oxidation in a shortcut to synthesize longer acyl chains (Figure 1; [28]). In ALB04 under inducing conditions, FabY and FabH3 are upregulated, while *pqsD* and *fabH2* are downregulated (Figure 4B). Therefore, SigX overexpression seems to be involved in the central FAS pathway and not in regulating specific compounds bearing fatty acid chains in their molecules. Interestingly, *fabH3* is the first gene in an operon containing the putative ECF sigma factor PA14_21550 and the putative anti-sigma PA14_21560 (Figure 6A), also induced in SigX overexpression, suggesting a sigma factor regulation cascade. PA14_21550, however, is not responsible for the upregulation of FAS genes, as shown in Figure 6.

*P. aeruginosa* PA14 also carries two *fabF* genes, but only the product of *fabF2* (PA14_46490) appears induced in SigX overexpression. *fabF2* codes for a β-oxoacyl-ACP-synthase II that may be regarded as an alternative isoform, since there is a paralogue (48% amino acid identity) which gene lies near *fabDG*, in an operon with several housekeeping genes and which mRNA levels were not changed in ALB04. It was reported that lack of FabF in a *fabB* thermosensitive *E. coli* mutant impairs the elongation of palmitoleic acid (C16Δ9) to cis-vaccenic acid (C18Δ11) [81]. Strains lacking FabB require unsaturated fatty acids for growth, suggesting that, *in vivo*, FabB must catalyze a key reaction in *E. coli* unsaturated fatty acid synthesis that FabF cannot accomplish [49]. FabB was also induced in ALB04 in the presence of arabinose at both protein and mRNA levels, therefore we cannot presume that the discrimination of FabB or FabF as a preferred synthase is regulated at expression levels by SigX.

We found that cells overexpressing SigX are swollen and have a more fluid membrane, according to anisotropy assays. The lower fluidity is probably due to the higher proportion of the FA with 16 carbon chains in ALB04 when compared to the control strain that presents more of the C18 FA (Figure 7A). There is little information in the literature about the control of FA chain length in *P. aeruginosa*. Zhu et al showed that the *trans-*2-enoyl-ACP reductases FabV and FabI can both act on substrates ranging from 2 to 16 C. However, whereas FabI activity is constant for all substrates, FabV activity increases with the substrate chain length up 12 C. The induction of FabV, which is already 10-fold more active than FabI [74], observed in our proteomic and qRT-PCR approaches, combined with the slight repression of *fabI*, may account for the higher proportion of shorter chain fatty acids in SigX overexpressing strain, contributing to the increased fluidity of the membrane. 

Even though the total lipid content of ALB04 cells is similar to ALB01 at four hours of SigX overexpression, when most of the former are still alive, we may speculate that the increased FAS enzymes may unbalance the lipid-to-protein ratio and cause cell death in later time points. However, some cells can overcome the deleterious impact of the stress caused by SigX overexpression and resume growth. It is well established for several sigma factors that their availability is auto-regulated. ECF factors are often sequestered by anti-sigma factors that are part of their own regulon. Other alternative sigma factors, such as σ^32^ and σ^s^, are regulated by proteolysis after the initial stress response (reviewed in Micevski and Dougan [82]). We found that SigX is downregulated in ALB04 at both mRNA and protein levels after several hours in the presence of arabinose (Figure 3); however, there is no current explanation for this fact. It does not seem to be a limitation of the overexpression system used, since PA14_21550 mRNA levels decrease much less than *sigX* in the same conditions. 

The data presented here show that SigX is involved in the regulation of FAS in the opportunistic pathogen *P. aeruginosa* and implies that this ECF sigma factor activates directly or indirectly an adaptive pathway that may contribute to alter membrane fluidity, resulting in the ability to resist to stresses that can disturb its integrity and interfere with cell survival. 

## Supporting Information

Figure S1 **Example of two-dimensional gels used at proteomic analysis of SigX overexpressing strain (ALB04) comparing to ALB01.** The spots highlighted are clearly induced in ALB04. The differential expression of these and the other spots was detected and confirmed after statistical analysis at Delta 2-D software (Decodon), as described in Material and Methods.
(TIF)

Table S1 **Oligonucleotides used in this work for cloning and qRT-PCR assays.**
(DOCX)

Table S2 **Additional proteins differentially expressed in SigX overexpressing strain.** The table shows the fold change in protein amounts in SigX overexpressing cells after 3h of arabinose induction in comparison to the control strain (ALB01) after 3h of arabinose induction, and the fold change in protein amounts in SigX overexpressing cells after 28h of arabinose induction in comparison to the control strain (ALB01) after 3h of arabinose induction.
(DOCX)
