# Supplementary figures and images for: A Novel Role for an ECF Sigma Factor in Fatty Acid Biosynthesis and Membrane Fluidity in Pseudomonas aeruginosa
Source: PLoS One. 2013 Dec 30;8(12):e84775. doi: 10.1371/journal.pone.0084775 (PMC3875570; doi:10.1371/journal.pone.0084775)

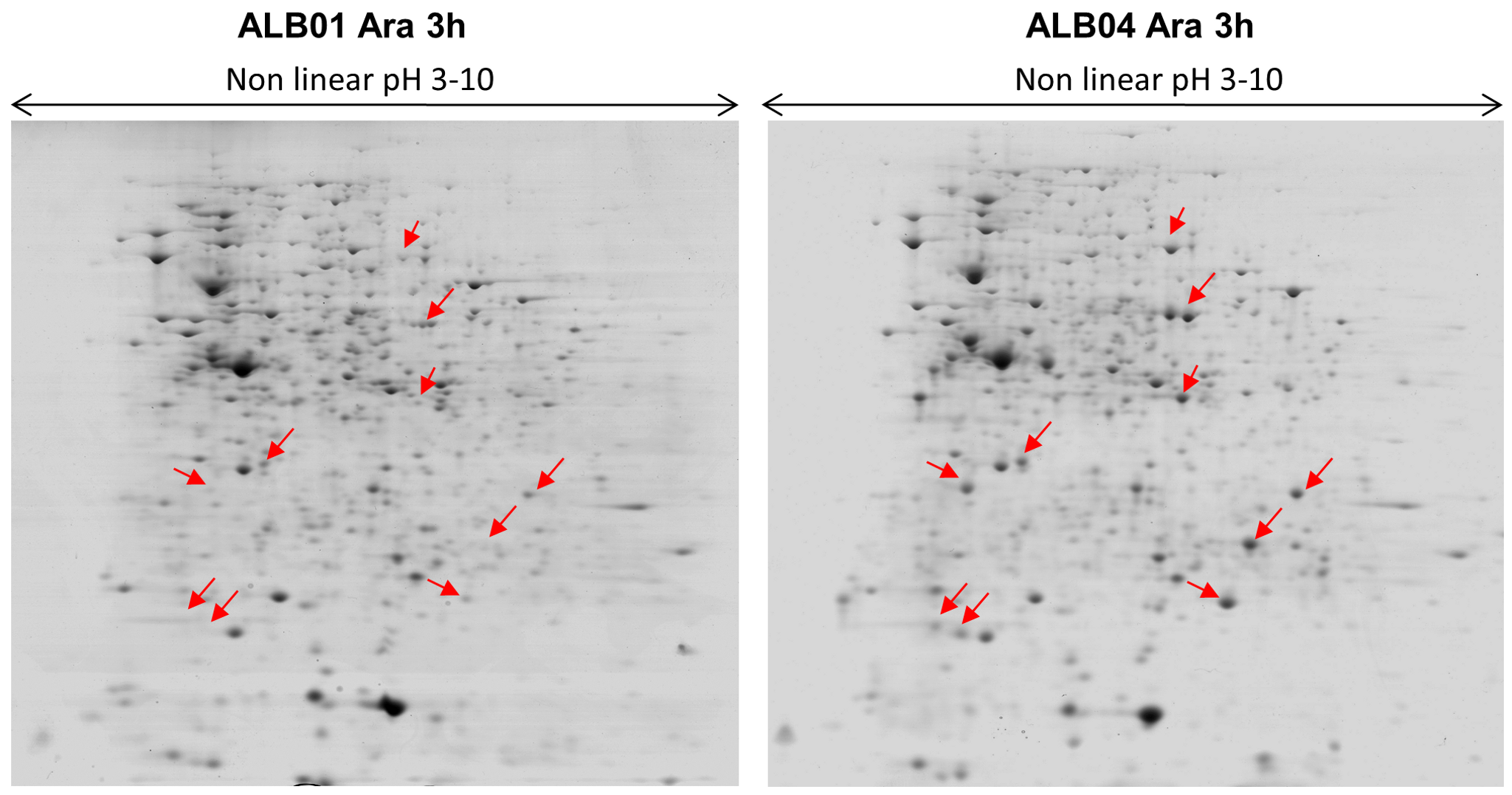

Supplement: Figure S1 — Example of two-dimensional gels used at proteomic analysis of SigX overexpressing strain (ALB04) comparing to ALB01. The spots highlighted are clearly induced in ALB04. The differential expression of these and the other spots was detected and confirmed after statistical analysis at Delta 2-D software (Decodon), as described in Material and Methods. (TIF) [file pone.0084775.s001.tif]
