# Supplementary material for: A Novel Role for an ECF Sigma Factor in Fatty Acid Biosynthesis and Membrane Fluidity in Pseudomonas aeruginosa
Source: PLoS One. 2013 Dec 30;8(12):e84775. doi: 10.1371/journal.pone.0084775 (PMC3875570; doi:10.1371/journal.pone.0084775)
Supplement: Table S1 — Oligonucleotides used in this work for cloning and qRT-PCR assays. (DOCX) [file pone.0084775.s002.docx]

**Table S1.** Oligonucleotides used in this work.

| ***Cloning^a^*** | |
| --- | --- |
| 21550_*Eco*RI | **GAATTC**GATCGGTAGCGTGATCC |
| 21550_*Spe*I | **ACTAGT**GTCCAGTTGCCCATCGAG |
| 41575_*Eco*RI | **GAATTC**GCCCGGCTTACGATATGA |
| 41575_*Pst*I | **CTGCAG**CATGTTGCGAACGCTGTC |
| *fabH3*_Ft_*Hin* | **AAGCTT**GGGCTGCTTCCTTGTGAA |
| *fabH3*_Ft_*Bam* | **GGATCC**GTACTGGCGCACGAAGGT |
| *sigX*_*Bam* | **GGATCC**GCCCGGCTTACGATATGA |
| *sigX*_*Eco* | **GAATTC**AGCCGATGACAACGCCTA |
| ***qRT-PCR*** | |
| *nadB* up | CTACCTGGACATCAGCCACA |
| *nadB* low | GGTAATGTCGATGCCGAAGT |
| *sigX*_qRT_L | CCAGGAGGTGATGCTGAAAG |
| *sigX*_qRT_R | CTGAGCGCATCCATCAATC |
| qRT_anicarb_L | AGAGCAGGAAAGCGCTGA |
| qRT_anicarb_R | TGGCTATTGGCGAGCTTC |
| 17270F | ACATCTTCGGCAATCTGTCC |
| 17270R | AGCCGATGTAGTCGAGGGTA |
| 64100F | ATGGTCGGCACCTTCTACC |
| 64100R | TCATCATCTTCATGGCTTCG |
| 64110F | GTGGAAGTCCAGGTGCTTTC |
| 64110R | GCCTCTTCGATCACCTTCTG |
| qRT_*accD*_L | CCTTCCATCATGCGTTCC |
| qRT_*accD*_R | ACGGTACAGCACGGCTTC |
| 25670F | GTGATGGCTCTGGAAGAGGA |
| 25670R | GCTGGTGAGCAACGATGTAG |
| qRT_*fabD*_L | CGTAGGGGCCAACTGATG |
| qRT_*fabD*_R | CACAGGCCGCCAATACAT |
| 68360F | GGTAACCGACGTGTTCATCA |
| 68360R | GGCTACCGTCAGGTAGTTGC |
| qRT_*fabH3*_L | CCAACAACATCCGCAACA |
| qRT_*fabH3*_R | GACCATCGGGCAGACATC |
| qRT_*fabG*_L | CGTTTCCAGCGACGAATC |
| qRT_*fabG*_R | GATGCCGGCGTTATTGAC |
| 57050F | GAAGCGCTGGAGAAGATGAC |
| 57050R | AAGATGTAGGCCACCGAATG |
| 43680F | GATCGCATCGTTCACATCAG |
| 43680R | GAAGTGACAGGCGAAGAACC |
| 17190F | CCGTTCTTCAACGGACATTT |
| 17190R | TCTTGAAACCGAGGATACCG |
| 25900F | GGAGCTGTGGGACAAGGTAA |
| 25900R | GGTTGAGGAACTCGCTCTTG |
| 41170F | AACATCAGCGCCTACAGCTT |
| 41170R | CAGGTAGGAGAGGGTCAGCA |
| 43690F | GACTCCGGAACAGATTTCCA |
| 43690R | ATGGCTTCCATCTGGTTCAG |
| 25690F | GACCATCAACCTGGACAACC |
| 25690R | AAACCGAACGAGTTCGACAG |
| 46490F | CCAGCGATTTCAACGACAG |
| 46490R | TTCCTCGATGACCAGTAGGC |
| 02620F | GAGGGCTACGGGATGAGC |
| 02620R | GTTGACGTTGGCCAGGTAGA |
| 51390F | TGCTGTACGGCTTGCAGA |
| 51390R | GGTCCGAACAGTCCATGC |
| qRT_fabHa_L | CGGGGTGGACACTTTCAG |
| qRT_fabHa_R | GCCTTCTTCCTCGGCATC |
| 10840F | TGGTGCTGATCTCCAGTGTT |
| 10840R | GTCTTCCCGTAGTGGACCAG |
| 10890F | CGGGAAGAACTGGACATCAC |
| 10890R | GTTGTTCACCAGCACATCCA |
| 23650F | GTATTCGCGACCAACGTGAC |
| 23650R | TCGAGGATACGTTGACGATG |
| 23950F | GAACGGTGGAGGATGTCG |
| 23950R | GTAGACCATTTTCCGGGTCA |
| 36870F | ACACGCTAAGGCAGAAATGG |
| 36870R | CACTGCTCTTCGCTGATGTC |
| 40900F | CATTCTCCAATCCCCCGTA |
| 40900R | ATAACCCTGTTGCCGAAGG |
| 45430F | ATGCCTGACATCACCCAAG |
| 45430R | CTGGTTGCTGGCATAGTTGA |
| 68040F | GGTGTTCAACGTCAACCTGT |
| 68040R | GCGATGGAGGTCACGTTTAT |
| 72880F | CGACATCCTGGTGAACAATG |
| 72880R | GACAGGGACAGTTGCCAATC |
| qRT_21550_L | TCATCGGCAGATCGCATA |
| qRT_21550_R | ATGGTCAGCAGCCAGGTC |

^a^ The restriction sites incorporated in the PCR products are in bold.
