# Supplementary material for: A Novel Role for an ECF Sigma Factor in Fatty Acid Biosynthesis and Membrane Fluidity in Pseudomonas aeruginosa
Source: PLoS One. 2013 Dec 30;8(12):e84775. doi: 10.1371/journal.pone.0084775 (PMC3875570; doi:10.1371/journal.pone.0084775)
Supplement: Table S2 — Additional proteins differentially expressed in SigX overexpressing strain. The table shows the fold change in protein amounts in SigX overexpressing cells after 3h of arabinose induction in comparison to the control strain (ALB01) after 3h of arabinose induction, and the fold change in protein amounts in SigX overexpressing cells after 28h of arabinose induction in comparison to the control strain (ALB01) after 3h of arabinose induction. (DOCX) [file pone.0084775.s003.docx]

**Table S2**. Additional proteins differentially expressed in SigX overexpressing strain.

| **Protein (gene)** | **Function^a^** | **ALB04 3h/ ALB01 3h^b^** | **ALB04 28h/ ALB013h^c^** |
| --- | --- | --- | --- |
| **Metabolic pathways other than fatty acid biosynthesis** | | | |
| PA14_29110 (*cysK*) | cysteine synthase A | 19.50 | 4.43 |
| PA14_23500 (*tyrB*) | aromatic amino acid aminotransferase | 2.09 | 1.06 |
| PA14_43970 (*lpdG*) | dihydrolipoamide dehydrogenase (operon *sucAB-lpdG;* Krebs cycle) | 2.05 | 1.35 |
| PA14_44010 (*sucA*) | 2-oxoglutarate dehydrogenase E1 component (operon *sucAB-lpdG;* Krebs cycle) | 1.84 | 0.4 |
| PA14_43940 (*sucD*) | succinyl-CoA synthetase subunit alpha (Krebs cycle) | 1.47 | 0.9 |
| PA14_43950 (*sucC*) | succinyl-CoA synthetase subunit beta (Krebs cycle) | 1.38 | 0.93 |
| PA14_09420 (*phzF1*) or PA14_39890 (*phzF2*) | phenazine biosynthesis protein PhzF | 0.64 | 1.68 |
| PA14_09450 (*phzD1*) or PA14_39925 (*phzD2*) | phenazine biosynthesis protein PhzD | 0.61 | 1.54 |
| **Regulatory function** | | | |
| PA14_08710 (*nusG*) | transcription antitermination protein NusG | 1.42 | 0.17 |
| PA14_22470 | LysR family transcriptional regulator | 0.67 | 2.04 |
| **Translation and protein metabolism** | | | |
| PA14_46950 | putative ATP-binding component of ABC transporter | 6.50 | 1.84 |
| PA14_41240 (*clpP*) | ATP-dependent Clp protease proteolytic subunit | 4.26 | 0.6 |
| PA14_27210 (*efp*) | elongation factor P | 2.15 | 1.21 |
| PA14_05450 | 16S ribosomal RNA methyltransferase RsmE | 1.63 | 4.29 |
| PA14_08830 (*tufA*) or PA14_08680 (*tufB*) | elongation factor Tu | 1.29 | 2.22 |
| PA14_57010 (*groEL*) | chaperonin GroEL | 1.21 | 4.02 |
| PA14_28650 (*thrS*) | threonyl-tRNA synthetase | 0.72 | 3.18 |
| PA14_08970 (*rplE*) | 50S ribosomal protein L5 | 0.67 | 2.53 |
| PA14_01710 (*ahpC*) | alkyl hydroperoxide reductase | 0.72 | 1.95 |
| PA14_53250 (*cbpD*) | chitin-binding protein CbpD | 0.61 | 4.38 |
| PA14_53070 (*hpd*) | 4-hydroxyphenylpyruvate dioxygenase | 0.72 | 1.82 |
| PA14_16250 (*lasB*) | elastase LasB | 0.90 | 3.68 |
| PA14_01760 (*nuh*) | nonspecific ribonucleoside hydrolase (gene close to *ahpC*) | 0.28 | 3.15 |
| **Hypothetical** | | | |
| PA14_11890 | hypothetical protein (Rossman fold: NAD(P) binding) | 3.22 | 5.63 |
| PA14_41730 | hypothetical protein | 0.50 | 2.0 |

^a^ As annotated [8,81,82]

^b^ fold change in protein amounts in SigX overexpressing cells after 3h of arabinose induction in comparison to the control strain (ALB01) after 3h of arabinose induction.

^c^ fold change in protein amounts in SigX overexpressing cells after 28h of arabinose induction in comparison to the control strain (ALB01) after 3h of arabinose induction.
